# Early-life risk factors for development of asthma from 8 to 28 years of age: a prospective cohort study

**DOI:** 10.1183/23120541.00074-2022

**Published:** 2022-12-12

**Authors:** Linnéa Hedman, Linnéa Almqvist, Anders Bjerg, Martin Andersson, Helena Backman, Matthew S. Perzanowski, Eva Rönmark

**Affiliations:** 1 Dept of Public Health and Clinical Medicine, Section of Sustainable Health, The OLIN Unit, Umeå University, Umeå, Sweden; 2 Martina Children's Hospital, Stockholm, Sweden; 3 Dept of Environmental Health Sciences, Mailman School of Public Health, Columbia University, New York, NY, USA; 4 These authors contributed equally to first authorship

## Abstract

**Background:**

The objective was to estimate the incidence rate of asthma from age 8 to 28 years and evaluate early-life risk factors for asthma onset at different ages.

**Methods:**

In 1996, within the Obstructive Lung Disease in Northern Sweden (OLIN) studies, a cohort of 3430 schoolchildren (97% of invited) was recruited at age 8 years to a prospective study about asthma. The cohort was followed annually from age 8 to 19 years and at 28 years by questionnaire surveys (67% of the original cohort participated). Asthma was categorised as never-asthma, onset age ≤8 years, onset age 9–13 years, onset age 14–19 years or onset age >19 years.

**Results:**

Of the 3430 individuals in the cohort, 690 (20.1%) reported asthma in any survey. The average incidence rate was 10.0/1000 per year at ≤8 years, 11.9/1000 per year at 9–13 years, 13.3/1000 per year at 14–19 years and 6.1/1000 per year at >19 years. The incidence was higher among boys until age 10 years, but from age 15 years, it became higher among girls. Family history of asthma, allergic sensitisation and breastfeeding <3 months were associated with asthma onset throughout the study. Low birthweight, maternal smoking during pregnancy, severe respiratory infection, rhinoconjunctivitis and eczema were associated with asthma onset ≤8 and 9–13 years.

**Conclusions:**

The incidence of asthma was high during childhood and the teenage period, and decreased substantially during young adulthood. Early-life factors were associated with asthma onset throughout childhood but had also a lasting effect on asthma incidence until adulthood.

## Abstract 2

**The incidence of asthma is high during childhood and teenage but decreases substantially during young adulthood. This study identified early-life risk factors for asthma in childhood; some remained important throughout teenage years and young adulthood.** https://bit.ly/3nxGNal

## Introduction

Asthma is a common disease with a particularly high incidence in childhood and the teenage years. Incidence rates ∼6–11/1000 per year have been reported in children and teenagers [1–4]. The incidence is also high among younger adults, ∼13/1000 per year [5], while the incidence is lower among older adults, ∼2–5/1000 per year [6, 7]. Among children, asthma is more common in boys than girls [4, 8–10], but in adolescence there is a gender shift, in part explained by increasing incidence among girls [3, 4, 10] and higher remission among boys [11].

The most important risk factors for asthma are a family history of asthma and allergic sensitisation [1, 9, 12–15]. Other risk factors include environmental exposures, such as environmental tobacco smoke (ETS) and house dampness, and in older individuals, occupational exposures and tobacco smoking [9, 12, 13, 16]. Several early-life risk factors have been suggested to have a long-lasting effect on asthma development, including low birthweight, short time of breastfeeding, severe respiratory infections, maternal smoking during pregnancy and postnatal ETS exposure [12, 13, 17–21]. Allergic rhinitis and eczema also often precede asthma development [22, 23].

However, the association between risk factors in early life and asthma has mostly been studied cross-sectionally in children or retrospectively in older ages and the findings are thereby subject to recall bias. There are only a few prospective studies about asthma where unselected, population-based cohorts have been followed from childhood up to adult age [8, 22–26]. Consequently, studies reporting the incidence of asthma from childhood to adulthood are particularly rare. Thus, the aim of this prospective cohort study was to estimate the incidence of physician-diagnosed asthma up to 28 years of age, and to study the association between early-life risk factors and asthma onset at different ages.

## Materials and methods

### Study design and sample

The study was performed within the Obstructive Lung Disease in Northern Sweden (OLIN) studies research programme. In 1996, OLIN's first paediatric cohort was recruited. The study design and recruitment procedure have been described previously [9]. In short, the parents of all children in first and second grade, aged 7–8 years (n=3525; median age 8 years) from three municipalities in northern Sweden were invited to participate in a questionnaire survey. Of those invited, 97% (n=3430) participated. The cohort was followed-up annually by a questionnaire until age 19 years. Of the original cohort, 2737 (79.8%) participated in at least 10 out of the 12 annual questionnaires. At age 28 years, a follow-up survey by postal questionnaire was conducted. Of the 3245 still alive and possible to trace, 2291 participated, constituting 71% of invited and 67% of the original cohort [25].

The Regional Ethical Review Board in Umeå, Sweden, approved the study. The parents and the participants (after they were aged 18 years) provided informed consent to participate in the study.

### Questionnaire

At all annual surveys from 8 to 19 years of age the same questionnaire was used: the International Study of Asthma and Allergies in Childhood protocol about asthma, rhinitis and eczema [27]. Additional questions about physician diagnosis of asthma and potential risk factors, such as parental smoking, respiratory infections and birthweight, were also included [28]. During the first years, the questionnaire was completed by the parents, but from 14 years of age the participants completed it themselves [29].

The follow-up at age 28 years was conducted with the OLIN questionnaire for adults, used within the OLIN studies since 1986 [30], and which has been compared with other commonly used questionnaires about asthma and allergic diseases [31]. The question about physician diagnosis of asthma was the same as in the previous annual questionnaires.

### Skin prick tests

The children in two of the municipalities were invited to skin prick tests (SPTs) at age 8, 12 and 19 years. 10 standard airborne allergens were included in the SPT: birch, timothy grass, mugwort, cat, dog, horse, two mites (*Dermatophagoides farinae* and *Dermatophagoides pteronyssinus*) and two moulds (*Cladosporium* and *Alternaria*) (Soluprick; ALK, Hørsholm, Denmark). A positive SPT was defined as a mean wheal ≥3 mm after 15 min to any of the 10 tested allergens.

### Definitions

Asthma was defined as an affirmative answer to the question “Has your child/have you been diagnosed by a physician as having asthma?”. At recruitment, the reported physician-diagnosed asthma was validated through structured interviews and clinical assessments by paediatricians [16]. Asthma at recruitment was defined as either a questionnaire report of physician-diagnosed asthma or as having asthma based on the clinical validation study [16].

Age at asthma onset was classified into mutually exclusive categories: asthma onset ≤8 years: onset before age 8 years, identified at recruitment (follow-up time: 7.5 years); asthma onset 9–13 years (follow-up time: 5 years); asthma onset 14–19 years (follow-up time: 6 years) based on reports of physician diagnosis of asthma in any of the annual questionnaire surveys. Asthma onset >19 years (follow-up time: 8.5 years) was based on reported physician diagnosis of asthma in the follow-up at age 28 years. The reference category “never-asthma” included those never reporting physician-diagnosed asthma in any of the questionnaire surveys.

Risk factors for asthma were based on parental questionnaire reports at recruitment at age 8 years, and included family history of asthma, maternal smoking during pregnancy, low birthweight, breastfeeding <3 months, any severe respiratory infection, rhinoconjunctivitis, eczema and allergic sensitisation. The definitions are described in detail in supplementary table E1.

### Statistical analyses

SPSS version 26.0 (IBM, Armonk, NY, USA) was used for analyses. The calculations for the statistical analyses are described in detail in supplementary material E2. In summary, the annual incidence rates between ages 8 and 19 years were calculated based on reports of asthma in the yearly questionnaires. For onset before age 8 years, the average annual incidence rate was calculated based on cases of asthma at recruitment. For onset after 19 years, the average annual incidence rate was calculated based on new cases of asthma in the questionnaire at age 28 years. A missing answer to the question about asthma was regarded as a negative response, while missing answers to questions about risk factors were regarded as missing and excluded from the analyses. The Chi-squared test was used for analysis of the association between potential risk factors and the age at asthma onset groups. A p-value of <0.05 was considered statistically significant. Significant or borderline significant risk factors (p<0.1) identified in the Chi-squared analyses were included in an adjusted multinomial regression analysis. The dependent variable was age at onset of asthma (categorical variable), with never-asthma as reference, and expressed as odds ratios with 95% confidence intervals.

## Results

### Incidence of asthma up to 28 years of age

Throughout the observation period, 690 (20.1%) of the entire cohort of 3430 individuals reported asthma in any of the surveys; in girls 20.8% and in boys 19.5% (p=0.338) (table 1). Of the 690 participants with asthma, 248 (35.9%) individuals had onset of asthma at ≤8 years of age, 169 (24.5%) at 9–13 years of age, 184 (26.7%) at 14–19 years of age and 89 (12.9%) at >19 years of age.

**TABLE 1:** Prevalence of ever-asthma^#^ from age 8 to 28 years and distribution by age at asthma onset and sex

	**All (n=3430)**	**Female (n=1679)**	**Male (n=1751)**	**p-value** ^¶^
**Ever-asthma**	690 (20.1)	349 (20.8)	341 (19.5)	0.338
**Age at asthma onset**				
≤8 years	248 (35.9)	105 (30.1)	143 (41.9)	0.061
9–13 years	169 (24.5)	78 (22.3)	91 (26.7)	0.547
14–19 years	184 (26.7)	116 (33.2)	68 (19.9)	<0.001
>19 years	89 (12.9)	50 (14.3)	39 (11.4)	0.156

Data are presented as n (%), unless otherwise stated. ^#^: a report of physician-diagnosed asthma in any of the questionnaire surveys; ^¶^: difference by sex analysed by the Chi-squared test.

**FIGURE 1:**
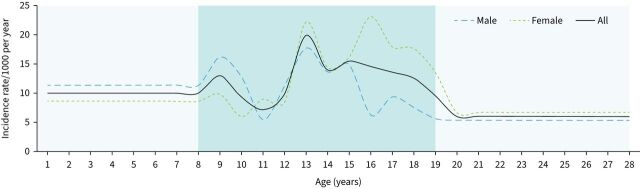
Incidence rates of asthma up to 28 years of age. The incidence rates before 8 and after 19 years, respectively, are average annual estimates based on reports of asthma at age 8 and 28 years, respectively. The incidence rates from 8 to 19 years are based on annual questionnaire reports of asthma.

The incidence rates of physician-diagnosed asthma are presented in figure 1. The average incidence rate was 10.0/1000 per year at ≤8 years, 11.9/1000 per year at 9–13 years, 13.3/1000 per year at 14–19 years and 6.1/1000 per year at >19 years. Until age 10 years, the annual incidence rate of asthma was in general higher among boys (average 13.4/1000 per year) than girls (average 8.2/1000 per year). From age 11 to 15 years, the incidence rate was similar in boys and girls, and tended to be highest between 12 and 14 years of age. From 15 to 19 years of age, the incidence rate was higher among girls (average 18.1/1000 per year) than boys (average 7.2/1000 per year). During young adulthood, the incidence rate decreased for both men and women, but was still higher among women (6.7/1000 per year) than men (5.4/1000 per year). The estimated average annual incidence rate of asthma from birth until 28 years of age was 8.0/1000 per year (7.7/1000 per year among men and 8.3/1000 per year among women).

### Risk factors in early life in relation to different ages at asthma onset

**FIGURE 2:**
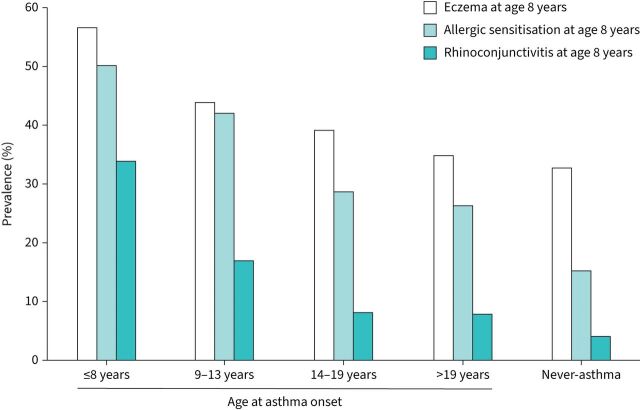
Prevalence of eczema, allergic sensitisation and rhinoconjunctivitis at age 8 years in relation to asthma development.

Compared with those who never reported asthma, those in any of the asthma onset categories were more likely to have a family history of asthma, low birthweight, breastfeeding <3 months, exposure to maternal smoking during pregnancy and any severe respiratory infection before 8 years of age (table 2). Maternal smoking during the first 2 years of life was slightly more common among those with asthma onset ≤8, 9–13 or 14–19 years compared with never-asthma, but without a statistically significant association. The prevalence of rhinoconjunctivitis, eczema and allergic sensitisation at age 8 years was the highest for asthma onset ≤8 years and decreased by increasing age at asthma onset (figure 2).

**TABLE 2:** Prevalence of potential risk factors in childhood among never-asthma and ever-asthma, and by different age at asthma onset

	**Never-asthma (n=2740)**	**Ever-asthma (n=690)**	**p-value** **^#^**	**Age at asthma onset**				**p-value** **^¶^**
				**≤8** **years (n=248)**	**9–13** **years (n=169)**	**14–19** **years (n=184)**	**>19** **years (n=89)**	
**Female sex**	1330 (48.6)	349 (50.6)	0.338	105 (42.3)	78 (46.2)	116 (56.2)	50 (56.2)	<0.001
**Family history of asthma**	470 (17.2)	252 (36.5)	<0.001	109 (44.0)	69 (40.8)	50 (27.2)	24 (27.0)	<0.001
**Low birthweight**	98 (3.7)	41 (6.2)	0.004	18 (7.6)	8 (4.9)	8 (4.5)	7 (8.0)	0.019
**Breastfeeding <3 months**	639 (24.0)	207 (31.2)	<0.001	88 (36.7)	28 (17.2)	59 (33.5)	32 (37.6)	<0.001
**Any severe respiratory infection before age 8 years**	1507 (55.0)	451 (65.4)	<0.001	191 (77.0)	103 (60.9)	107 (58.2)	50 (56.2)	<0.001
**Smoking during pregnancy**	646 (24.0)	196 (29.3)	0.005	74 (30.2)	47 (29.0)	55 (30.9)	20 (23.5)	0.043
**Exposure to maternal smoking in first 2 years of life**	816 (31.1)	228 (34.8)	0.075	85 (35.7)	53 (33.3)	64 (36.6)	26 (31.0)	0.374
**Exposure to maternal smoking at age 8 years**	809 (30.3)	241 (36.2)	0.003	94 (39.3)	58 (35.8)	59 (33.0)	30 (35.3)	0.030

Data are presented as n (%), unless otherwise stated. Missing values in individual variables: low birthweight n=124, breastfeeding n=104, smoking during pregnancy n=65, exposure to maternal smoking in first 2 years n=94 and exposure to maternal smoking at age 8 years n=153. ^#^: difference between never-asthma and ever-asthma analysed by the Chi-squared test. ^¶^: difference between never-asthma and all asthma onset groups analysed by the Chi-squared test.

### Risk factors related to different ages at asthma onset assessed by multinomial regression analyses

Female sex was a protective factor against asthma onset ≤8 years and a risk factor for asthma onset 14–19 years (table 3). Family history of asthma was consistently significantly associated with all asthma categories, with the highest odds ratio for asthma onset ≤8 years. Breastfeeding <3 months was associated with all asthma onset categories. Low birthweight, any severe respiratory infection and eczema were associated with asthma onset ≤8 years. Maternal smoking during pregnancy was associated with asthma onset 9–13 years.

**TABLE 3:** Associations (odds ratio (95% confidence interval)) between risk factors in childhood and asthma by age at onset^#^

	**Age at asthma onset**			
	**≤8 years**	**9–13 years**	**14–19 years**	**>19 years**
**Female sex**	0.74 (0.54–0.99)	0.93 (0.66–1.29)	1.84 (1.33–2.56)	1.22 (0.78–1.91)
**Family history of asthma**	3.30 (2.43–4.48)	3.25 (2.31–4.57)	1.55 (1.08–2.24)	2.04 (1.25–3.34)
**Low birthweight**	1.87 (1.04–3.36)	1.24 (0.55–2.78)	1.15 (0.54–2.44)	2.12 (0.94–4.82)
**Breastfeeding <3 months**	2.02 (1.45–2.81)	0.63 (0.40–0.98)	1.57 (1.10–2.23)	1.75 (1.07–2.85)
**Smoking during pregnancy**	1.30 (0.93–1.84)	1.54 (1.05–2.25)	1.33 (0.93–1.90)	0.78 (0.45–1.35)
**Any severe respiratory infection**	2.76 (1.95–3.90)	1.31 (0.93–1.84)	1.11 (0.80–1.52)	1.06 (0.68–1.67)
**Rhinoconjunctivitis**	6.55 (4.47–9.61)	2.78 (1.68–4.59)	1.48 (0.80–2.74)	1.50 (0.61–3.65)
**Eczema**	1.75 (1.29–2.37)	1.30 (0.93–1.83)	1.20 (0.86–1.66)	0.87 (0.53–1.41)
**Allergic sensitisation**	3.20 (2.16–4.74)	2.92 (1.87–4.55)	2.23 (1.45–3.43)	1.95 (0.97–3.94)

^#^: analysed in a multivariable multinomial regression analysis, with never-asthma as reference.

Rhinoconjunctivitis at age 8 years was associated with asthma onset ≤8 and 9–13 years. Allergic sensitisation at 8 years was significantly associated with all categories of age at asthma onset, with the highest odds ratio for ≤8 years. The analysis was also performed by adjusting for allergic sensitisation at age 8, 12 or 19 years and yielded similar results (supplementary table E3).

## Discussion

In this population-based, prospective study of asthma, in an unselected cohort of 3430 children followed from 8 to 28 years of age, we found that the incidence of asthma was highest in childhood and the teenage years, and decreased in young adulthood. The incidence was higher among boys until the age of 10 years, but from 15 years, it became higher among girls. Several risk factors in early life or early childhood were still of importance for the incidence of asthma in young adulthood, including short time of breastfeeding, family history of asthma and allergic sensitisation, while low birthweight, maternal smoking during pregnancy, severe respiratory infections and eczema mainly were associated with onset of asthma in childhood and adolescence. Rhinoconjunctivitis at age 8 years associated strongly with asthma development for all age at onset groups.

Until 10 years of age, the incidence of asthma varied between 9 and 13/1000 per year, with higher rates among boys than girls, in accordance with other studies [2, 6]. After a plateau with similar asthma incidence rates, the rates increased and peaked during the early teenage years in both boys and girls. Our detailed annual examinations clearly demonstrate the shift from male to female dominance after 15 years of age. The increased incidence of asthma among girls and higher remission rates among boys [11] contribute to the higher prevalence of asthma among women in adults [10, 32].

Overall, studies on asthma incidence from childhood until young adulthood with multiple follow-ups are scarce. Both studies from the 1990s and more recent ones, including our study, show incidence rates of ∼6–11/1000 per year [2, 3, 7, 8, 33]. However, comparing the incidence rates between studies is difficult for many reasons. First, some of the available prospective studies recruited their participants during the 1950–1970s [8, 22, 26], and the prevalence, treatment and awareness of asthma has changed considerably since then [32, 33]. Second, there are different methods to calculate the incidence as it can be based either on prospective or retrospective studies [34, 35] and, as clearly shown in the current study, the age of the study population is of importance. Moreover, the incidence of asthma during adolescence may be underestimated if the times of baseline and end-point measurements are too far apart [4]. Third, the definition of the outcome variable may yield different results. In epidemiological studies asthma can, for instance, be defined based on respiratory symptoms, use of asthma medication, different measures of bronchial airflow variability or physician diagnosis. We chose physician-diagnosed asthma as outcome as it has higher specificity compared with symptom-based variables [16]. However, this may underestimate the real incidence due to undiagnosed asthma.

Risk factors may have different impacts depending on both age at exposure and age at onset of asthma. In this study, we found that the association with family history of asthma was strongest for asthma with onset in childhood, but still important for onset during the teenage years and young adulthood. This is in line with a large Danish twin study reporting that genetic factors were associated with asthma throughout the life span, while the importance of heritability decreased with increasing age [36]. That the strength of association decreased with increasing age at asthma onset may be a delusion effect where those most susceptible develop asthma early in life. It has been suggested that the heritability of asthma is ∼60–70% [13, 36], although the genetic and environmental interactions are not yet fully understood. It could be hypothesised that some gene–environment interactions are less associated with parental asthma in childhood, but may become more important in adulthood due to new environmental and lifestyle exposures, such as tobacco smoking and occupational exposures [12, 13].

The progression of allergic diseases usually starts with the development of food allergy and eczema followed by allergic sensitisation. A suggested explanation would be that a defective skin barrier, skin inflammation and/or microbiome alterations could promote the development of sensitisation [37, 38]. Allergic sensitisation is, in turn, one of the strongest predictors of asthma and allergic rhinitis, and usually precedes these conditions. We found that allergic sensitisation at age 8 years was associated with asthma onset in childhood and the teenage years as well as borderline associated with asthma in young adulthood. Moreover, we found that rhinoconjunctivitis at age 8 years was associated with subsequent asthma onset, in line with other prospective studies [22, 39]. Although the temporal sequence of allergic diseases was not fully investigated in the current article, in another publication based on this cohort it was clearly shown that particularly early sensitisation was strongly associated with the development of both asthma and rhinitis during adolescence [40].

In line with previous studies, respiratory infections in early life were associated with asthma in childhood [12, 13, 20]. It has been shown that both severe symptoms of respiratory syncytial virus (RSV) and the number of early respiratory infections, including both RSV and rhinovirus, were associated with subsequent asthma development [12, 20]. Thus, primary prevention of severe respiratory infections, particularly RSV during the first years of life, is important. Previous analyses of our cohort showed that respiratory infections were associated only with nonallergic asthma [16] and that infections had no impact on allergic sensitisation [28], the latter in line with other studies [41, 42]. Even though exclusive or prolonged breastfeeding could reduce infections and early viral wheezing [13, 19], the long-term effects on asthma are unclear [19, 41, 42]. In the current study, short time of breastfeeding was associated with asthma incidence up to young adulthood, suggesting a long-term protective effect of breastfeeding. However, the opposite has also been reported [41], indicating the complexity of the topic as both short time of breastfeeding and respiratory infections are more common in children with smoking mothers, which complicates the analyses.

In our study, we found that maternal smoking during pregnancy was associated with asthma, particularly with onset 9–13 years. It has been shown that maternal smoking during pregnancy and/or during infancy increased the risk for wheezing and asthma, and had a negative effect on lung function development throughout childhood [21, 24]. Moreover, smoking during pregnancy is also associated with low birthweight, which in turn is associated with asthma [17, 18]. Thus, the interaction between tobacco smoke exposure, low birthweight, breastfeeding and respiratory infections may have long-term effects on lung function and asthma throughout childhood and adolescence, and could partly explain the significant association between breastfeeding and onset of asthma in young adulthood in our study.

The strengths of our study are the prospective study design with long-term follow-up and high participation and retention rates throughout the study period. The change from parental to self-completed questionnaires has previously been evaluated and showed excellent agreement, especially regarding the question about physician diagnosis of asthma [29]. A limitation was that the asthma diagnosis was not clinically verified throughout the whole observation period. However, at recruitment, the asthma diagnosis was validated, showing >99% specificity and >70% sensitivity for parental-reported physician-diagnosed asthma [16]. Incident asthma cases during the pre-teen and teenage period were clinically verified by spirometry with reversibility tests or bronchial variability (unpublished data), while cases from age 19 to 28 years have not been clinically verified. Because questions about exposures in early life were completed by the parents at recruitment, the risk of recall and reporting bias was reduced. A limitation was that parental socioeconomic status was not included in any of the surveys.

In conclusion, in this prospective cohort study, we found that the incidence of asthma was high in childhood and the teenage years, and decreased in young adulthood. In childhood, the incidence rates were higher among boys, but after 15 years of age rates were higher among girls. Several early-life risk factors for asthma in childhood were identified and many of them remained important throughout the teenage years and young adulthood. Prevention of respiratory infections and ETS and promotion of breastfeeding may reduce not only childhood asthma but also onset in young adulthood.

## Supplementary material

10.1183/23120541.00074-2022.Supp1 **Please note:** supplementary material is not edited by the Editorial Office, and is uploaded as it has been supplied by the author.
Supplementary material 00074-2022.SUPPLEMENT
