# Supplementary material for: Early-life risk factors for development of asthma from 8 to 28 years of age: a prospective cohort study
Source: ERJ Open Res. 2022 Dec 12;8(4):00074-2022. doi: 10.1183/23120541.00074-2022 (PMC9835990; doi:10.1183/23120541.00074-2022)
Supplement: Supplementary file 1 [file 00074-2022.SUPPLEMENT.pdf]

Online data supplement

Table E1. Definition of risk factors for asthma based on parental questionnaire reports at recruitment at age 8y

Definition	Explanation
Family history of asthma	Mother or father with asthma
Maternal smoking during pregnancy	An affirmative answer to the question "Did the mother smoke during pregnancy?"
Low birthweight	Birthweight <2500 gram
Any severe respiratory infection	A history of pertussis, croup, pneumonia or other severe respiratory infection.
Rhinoconjunctivitis	Affirmative answer to the questions "In the past 12 months, has your child had a problem with sneezing, or a runny, or a blocked nose when he/she did not have a cold or the flu?" and "In the past 12 months, has this nose problem been accompanied by itchy-watery eyes?"
Eczema	Affirmative answer to the question: "Has your child ever had eczema?"
Allergic sensitization	A positive skin prick test at age 8 years, and for an additional analysis, any positive skin prick test at age 8, 12 or 19 years.

E2. Calculations of incidence rate and early life risk factors

Incidence rate

The incidence rate was calculated as: $\frac{a}{Followup\ time \times (b-(a/2)}\times 1000$ where *a* is the incident cases and *b* the population at risk. The incident cases of asthma each year were excluded from the population at risk for the calculation of incidence rate in the next year. Non-participants in individual surveys did not contribute with person-years that specific survey.

The calculation of the average annual incidence rate from birth to 8y was based on the questionnaire survey and clinical assessment at 8y and the average follow-up time was 7.5 years. The onset of asthma was set at midpoint between birth and 8y, i.e. at 3.75 years.

The questionnaire surveys between 8 and 19 years of age were performed with yearly intervals, thus the follow-up time between each survey was one year. The onset of asthma was set at midpoint between surveys, 0.5 years.

We also calculated the average incidence rate for different age intervals as the mean of the yearly incidence rates.

The average incidence rate of asthma between 19 and 28y was based on the questionnaire survey at 28y and the average follow-up time was 8.5 years, thus the midpoint was 4.25 years.

Multinomial regression analysis of early life risk factors for asthma with onset at different ages

Early life risk factors for asthma with onset at different ages were analysed in an adjusted multinomial regression analysis. For the dependent variable, we used the entire cohort of 3430 individuals, even if not all contributed with data in all surveys. We chose a ‘last observation carried forward’ approach, and assumed that individuals that did not participate remained in the ‘never asthma’ category if they had not already been defined into any of the asthma categories.

Table E3. The association between risk factors in childhood and asthma by age at onset. Analyzed in a multivariable multinomial regression analysis, with 'never asthma' as reference and presented as odds ratios (OR) with 95% confidence intervals (95% CI). Allergic sensitization either at age 8y, 12y or 19y was included in the analysis.

	Age at asthma onset							
	≤8 years		9 to 13 years		14 to 19 years		>19 years	
	OR	95% CI	OR	95% CI	OR	95% CI	OR	95% CI
Female sex	0.57	(0.41-0.79)	1.07	(0.78-1.47)	1.72	(1.25-2.36)	1.28	(0.82-1.99)
Family history of asthma	3.16	(2.27-4.41)	3.09	(2.23-4.27)	1.57	(1.10-2.25)	1.98	(1.22-3.20)
Low birthweight	2.07	(1.10-3.88)	1.36	(0.65-2.82)	1.07	(0.50-2.27)	2.42	(1.11-5.25)
Breastfeeding <3 months	1.97	(1.37-2.84)	0.71	(0.47-1.08)	1.65	(1.17-2.33)	1.90	(1.18-3.06)
Smoking during pregnancy	1.26	(0.86-1.84)	1.63	(1.14-2.33)	1.32	(0.93-1.87)	0.76	(0.44-1.30)
Any severe respiratory infection	2.71	(1.86-3.96)	1.42	(1.03-1.98)	1.13	(0.83-1.55)	1.02	(0.66-1.59)
Rhinoconjunctivits	5.73	(3.81-8.62)	4.02	(2.62-6.16)	1.92	(1.11-3.32)	1.82	(0.80-4.14)
Eczema	1.64	(1.18-2.29)	1.40	(1.01-1.93)	1.10	(0.80-1.53)	0.89	(0.55-1.43)
Allergic sensitization*	3.43	(2.28-5.14)	3.17	(2.15-4.67)	2.31	(1.61-3.32)	1.44	(0.84-2.45)

*Including the n=2443 that participated in skin prick test at age 8y, 12y or 19y.
